# Leaf traits divergence and correlations of woody plants among the three plant functional types on the eastern Qinghai-Tibetan Plateau, China

**DOI:** 10.3389/fpls.2023.1128227

**Published:** 2023-04-03

**Authors:** Hongshuang Xing, Zuomin Shi, Shun Liu, Miao Chen, Gexi Xu, Xiangwen Cao, Miaomiao Zhang, Jian Chen, Feifan Li

**Affiliations:** ^1^ Key Laboratory of Forest Ecology and Environment of National Forestry and Grassland Administration, Ecology and Nature Conservation Institute, Chinese Academy of Forestry, Beijing, China; ^2^ Miyaluo Research Station of Alpine Forest Ecosystem, Lixian, China; ^3^ Co-Innovation Center for Sustainable Forestry in Southern China, Nanjing Forestry University, Nanjing, China

**Keywords:** plant functional type, leaf trait, elevation, climate, survival strategy

## Abstract

Leaf traits are important indicators of plant life history and may vary according to plant functional type (PFT) and environmental conditions. In this study, we sampled woody plants from three PFTs (e.g., needle-leaved evergreens, NE; broad-leaved evergreens, BE; broad-leaved deciduous, BD) on the eastern Qinghai-Tibetan Plateau, and 110 species were collected across 50 sites. Here, the divergence and correlations of leaf traits in three PFTs and relationships between leaf traits and environment were studied. The results showed significant differences in leaf traits among three PFTs, with NE plants showed higher values than BE plants and BD plants for leaf thickness (LT), leaf dry matter content (LDMC), leaf dry mass per area (LMA), carbon: nitrogen ratio (C/N), and nitrogen content per unit area (N_area_), except for nitrogen content per unit mass (N_mass_). Although the correlations between leaf traits were similar across three PFTs, NE plants differed from BE plants and BD plants in the relationship between C/N and N_area_. Compared with the mean annual precipitation (MAP), the mean annual temperature (MAT) was the main environmental factor that caused the difference in leaf traits among three PFTs. NE plants had a more conservative approach to survival compared to BE plants and BD plants. This study shed light on the regional-scale variation in leaf traits and the relationships among leaf traits, PFT, and environment. These findings have important implications for the development of regional-scale dynamic vegetation models and for understanding how plants respond and adapt to environmental change.

## Introduction

Plant functional traits are useful tools for exploring how plants adapt to the environment and studying global climate change (Díaz et al., 2016; Fyllas et al., 2020; Cano-Arboleda et al., 2022). Among these traits, leaf traits have received particular attention due to their sensitivity to climate change and their ability to reflect plant resource acquisition and utilization (Wilson et al., 1999; Meng et al., 2015; Baruch et al., 2017; Ye et al., 2022). In dry conditions, plants tend to have thicker leaf thickness (LT), higher leaf dry mass per area (LMA), and larger leaf dry matter content (LDMC), in order to reduce water loss and enhance their ability to adapt to the drought environments (Akram et al., 2020; Akram et al., 2022). Leaf nitrogen content is closely related to photosynthesis (Chen et al., 2013; Zhan et al., 2018). The leaf carbon capture strategy can be represented by nitrogen content per unit area (N_area_), nitrogen content per unit mass (N_mass_), and carbon: nitrogen ratio (C/N) (Chen et al., 2013; Zhan et al., 2018). Plants typically had higher N_area_ and LMA under hot and dry environmental conditions, as this increased investment of nitrogen in structure enhanced their survival in adversity (Wright et al., 2003; Blumenthal et al., 2020; Himes et al., 2020). As essential members of plant functional traits, leaf traits can provide insight into the relationship between plants and the environment at both the regional and global scales (Dong et al., 2020; Xu et al., 2021; Toledo-Aceves et al., 2022).

The interrelationships among leaf traits can be affected by historical contingencies and current environmental pressures, and the relationships between leaf traits can differ among different plant functional types (PFTs) (Garnier et al., 2001; Liu et al., 2008; Jones et al., 2013; Díaz et al., 2016; Fyllas et al., 2020). The research found that LMA and LDMC were correlated with LT, and the thicker leaves showed a trade-off between higher leaf toughness (physical strength) and lower leaf photosynthetic rate (Wilson et al., 1999; Pérez-Harguindeguy et al., 2013). Although general dimensions of variation in leaf traits had been observed worldwide to determine basic plant survival strategies, but recent studies had shown that the relationship might be unstable on a local scale and there were difference among different PFTs (Fyllas et al., 2020; Chen et al., 2023). The comparative study of leaf trait variation among different PFTs is helpful to determine the plant survival strategy and parameterization of dynamic vegetation models (Adler et al., 2014). According to leaf habit and form, woody plants can be divided into needle-leaved evergreen (NE) woody plants, broad-leaved evergreen (BE) woody plants and broad-leaved deciduous (BD) woody plants (Fyllas et al., 2020). NE plants can survive in colder environments due to their relatively high cavitation resistance and nutrient use efficiency (Bond, 1989; Brodribb et al., 2012). BE plants and NE plants generally have the longer leaf life span, and they can survive and maintain photosynthesis during a long period of soil water deficit (Box and Fujiwara, 2015). BD plants are more competitive than NE plants and BE plants under the conditions of adequate moisture due to higher photosynthetic capacity and hydraulic conductivity, which placed them toward the ‘acquisitive’ part of the leaf economic spectrum (Wright et al., 2004; Berendse and Scheffer, 2009).

The Qinghai-Tibet Plateau provides natural experimental sites for the study of leaf traits, as it has a large elevation drop, high species richness, and complex community structure (Kang et al., 2021). However, under the context of multi-level changes in the alpine environment caused by climate change, the study is not very comprehensive that the response of leaf traits to environmental change among different PFTs in this region. It is essential to understand variation in leaf traits among different PFTs and response and adaption of plant to environmental change. This study mainly focused on woody plants on the eastern Qinghai-Tibetan Plateau, and objectives were (1) to understand the variation in leaf traits among different PFTs, (2) to explore the change patterns and relationships in leaf traits among different PFTs in the subalpine environment, and (3) to clarify the relationship among leaf traits, PFTs, and the environment (elevation and climate).

## Materials and methods

### Research sites

**Figure 1:**
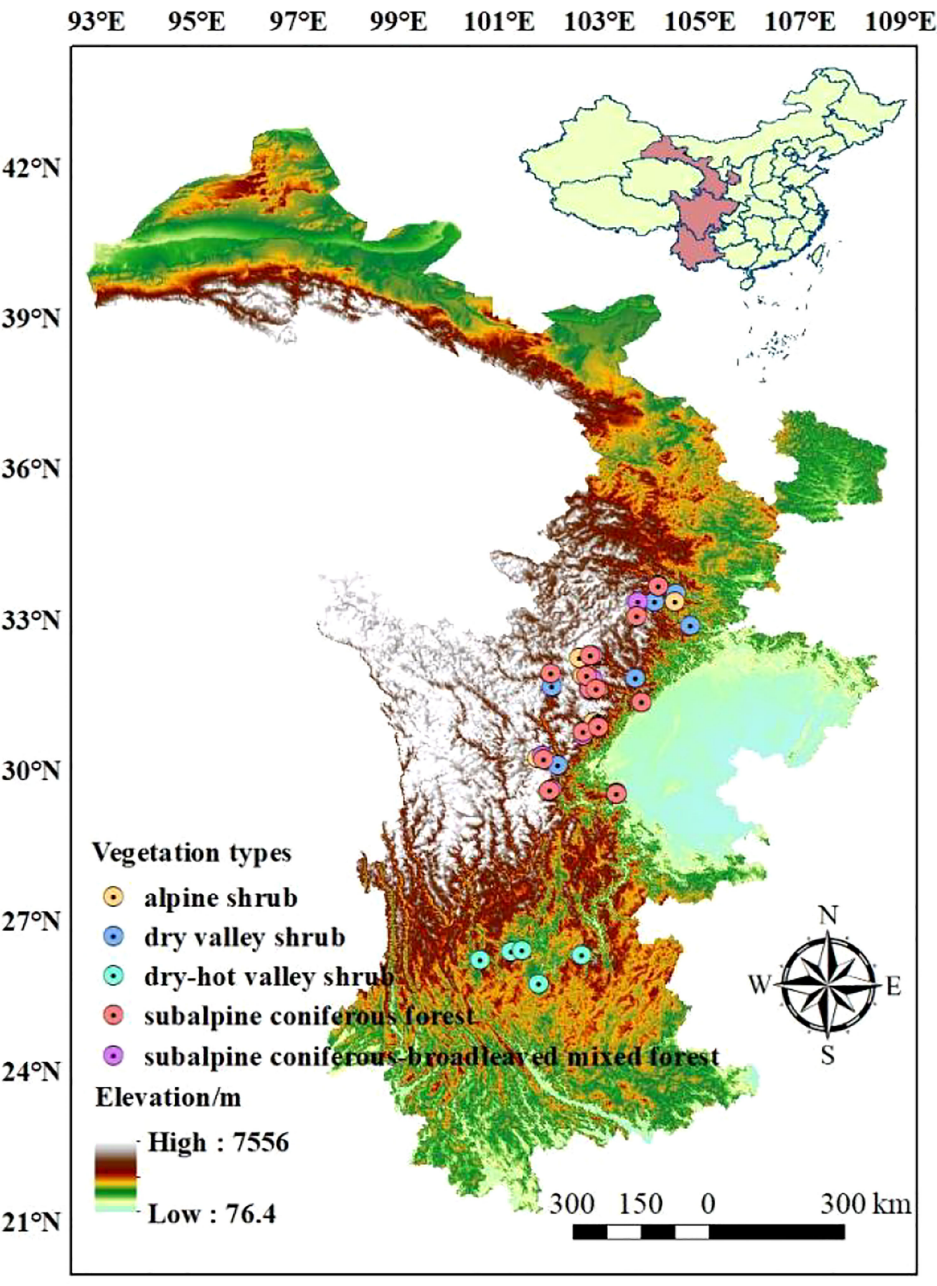
Geographic locations of the research sites.

The Qinghai-Tibetan Plateau boasts a unique natural environment and spatial differentiation, which is attributed to the reduction of atmospheric circulation and the distinct topography of the plateau (Li et al., 2022). The distinctive geographical combination of water and thermal conditions created ideal experimental sites for this research. The research sites were primarily situated in the eastern Qinghai-Tibetan Plateau, across the Yunnan, Sichuan, and Gansu provinces of China (25.72 °N - 33.67 °N, 98.49 °E - 104.82 °E). The mean annual precipitation (MAP) at the research sites ranged from 525 mm to 1240 mm, and the mean annual temperature (MAT) ranged from -4 °C to 21 °C. Additionally, the sites contained a large elevation drop (860 m - 4200 m) and an array of vegetation types, including alpine shrub, subalpine coniferous forest, subalpine coniferous and broad-leaved mixed forest, dry valley shrub, and dry-hot valley shrub (Du et al., 2020). These natural experimental conditions provided an excellent basis for our investigation into the leaf traits of woody plants (**Figure 1**; **Table S1**).

### Field sampling

Field surveys and sampling were conducted from 2018 to 2020 during the peak period for plant growth (July to September). The 50 broadly representative woody sample sites on the eastern Qinghai-Tibetan Plateau were selected and 3 - 5 plots were set up (forest, 20 m × 20 m; shrub, 10 m × 10 m) at each site. To minimize human interference, the plots were set up within natural reserves, and we recorded the longitude and latitude of each plot using a global positioning system (Thales Navigation, Santa Barbara, CA, USA). Five to seven individuals of each species with 15 - 20 g leaves per individual within a plot were collected as one sample for measuring leaf traits. Expanded, mature, and sun-exposed leaves of trees and shrubs were collected. Totally, the 771 samples were collected from 110 species across the 50 sites, including three plant functional types (PFTs) (e.g., needle-leaved evergreens, NE; broad-leaved evergreens, BE; and broad-leaved deciduous, BD).

### Leaf traits measurement

The study measured six leaf traits, including leaf thickness (LT, mm), leaf dry matter content (LDMC, mg/g), leaf dry mass per area (LMA, mg/cm^2^), nitrogen content per unit area (N_area_, mg/cm^2^), nitrogen content per unit mass (N_mass_, mg/g), and carbon:nitrogen ratio (C/N) (Rawat et al., 2021). LT was measured using a vernier caliper with an accuracy of 0.01 mm, and the leaf fresh weight (LFW) was weighed using an electronic balance with an accuracy of 0.001 g. The leaf area (LA) was calculated using image analysis software (image J v.1.8.0) after scanning the leaves using a scanner (Seiko Epson Co., Nagano, Japan). After recording all the measurements, the leaf samples were baked in an oven at 75°C for 72 hours to a constant weight, and the leaf dry weight (LDW) was weighed. N_mass_, N_area_, and C/N were analyzed after the leaf samples were ground into fine powder using a steel ball mixing mill MM200 (Retsch GmbH, Haan, Germany) (Cornelissen et al., 2003; Pérez-Harguindeguy et al., 2013). LMA and LDMC were calculated using the following equations:


$$\text{LMA}=\frac{\text{LDW}}{\text{LA}}$$

$$\text{LDMC}=\frac{\text{LDW}}{\text{LFW}}$$

### Climate data

Meteorological data for the study was obtained from China Ecosystem Research Network. The dataset was generated using data from 2400 weather stations of the China Meteorological Administration spanning from 1980 to 2020 and had a spatial resolution of 1 × 1 km (https://data.cma.cn). MAT and MAP values for each plot were extracted using the interpolation software of ANUSPLIN (v.4.36) (Hutchinson and Xu, 2013) from climate dataset. This allowed us to obtain accurate and representative climatic conditions of the study area.

### Data analysis

The data analyzed in this study include species-mean trait values for each species sampled at each plot (Dong et al., 2020; Wang et al., 2022). Linear mixed models were used to explore whether the variation of leaf traits was independent of PFTs identity. In this analysis, species were used as fixed effects and PFTs were used as random effects (package Ime4, Ismeans). LSD’s *post-hoc* test was then performed to compare the differences in leaf traits among three PFTs. Principal component analysis (PCA) was utilized to explore differences in major functional dimensions among three PFTs. Linear models and Pearson’s correlation were applied to determine relationships between leaf traits. Linear models were used to explore whether the effect of environmental variability on leaf traits was independent of PFTs. At the same time, linear mixed models with PFTs as random effects and environmental factors as fixed effects were used to compare and verify the results of the linear model. Because there was no obvious difference between the results of the two models, the results of the linear model were finally selected for analysis (Warton et al., 2006). To quantify the relative contributions of PFTs, MAT, and MAP on leaf traits, partial general linear models analyses were used with leaf traits as dependent variables and PFTs, MAT, and MAP as predictors. The partial regressions was used to divide the variation in response variables explained by predictive variables into independent components (PFTs, MAT, MAP) and joint components (PFTs - MAT, PFTs - MAP, MAT - MAP, PFTs - MAT - MAP) (Zhan et al., 2018). Additionally, linear mixed models and linear models were conducted using R v.3.6.1 (R Core Team, 2019), the other analyses were performed using SPSS v.23.0 (Field, 2013), the graphs were performed using Origin v.2021 (Chen, 2006).

## Results

### Leaf traits of different PFTs

**Figure 2:**
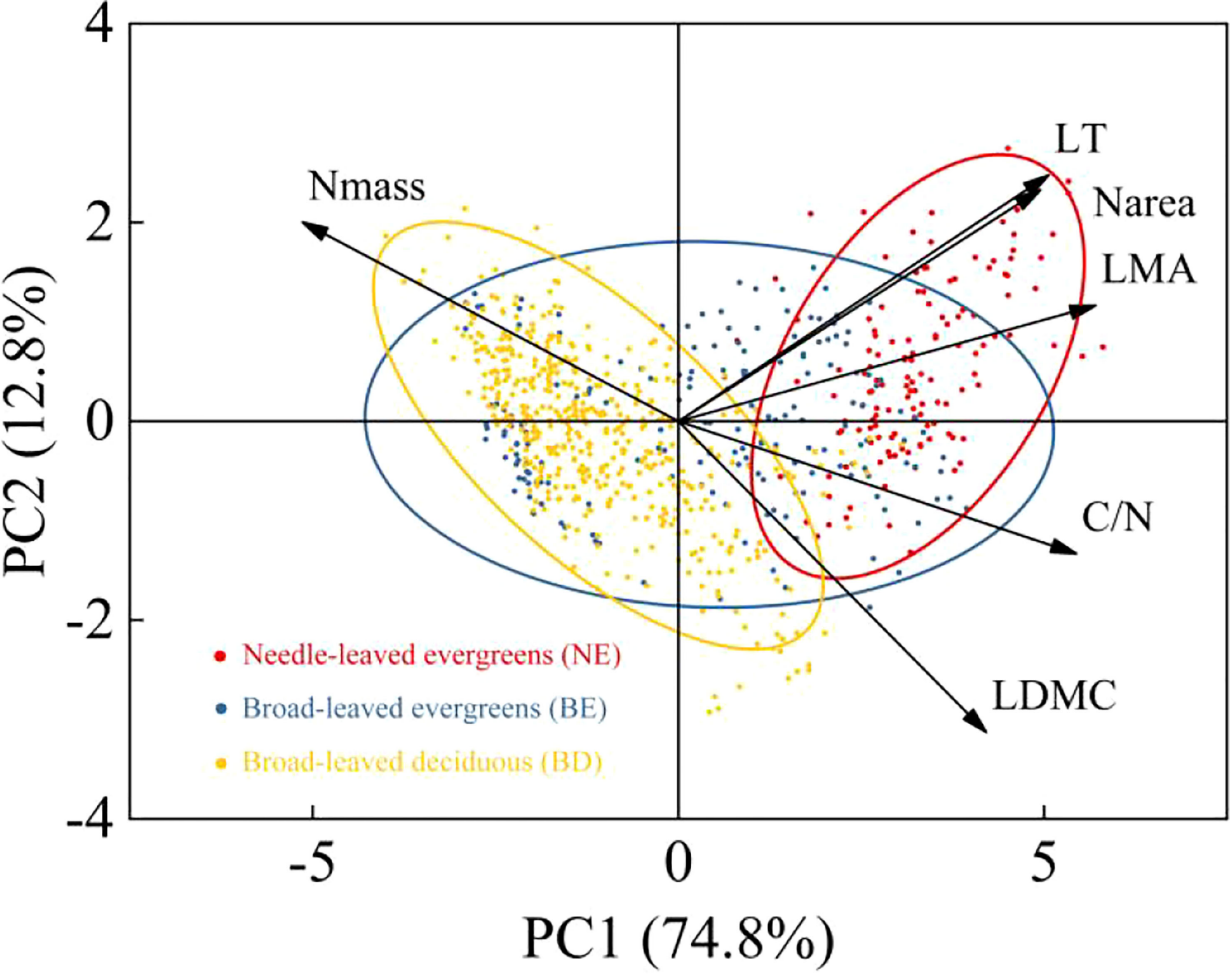
Principal components analysis of six leaf traits.

The result indicated that there were significant differences in leaf traits among different PFTs (p<0.01) (**Table 1**). LT, LDMC, LMA, C/N, and N_area_ of NE were significantly higher than those of BE and BD, and showed the pattern of NE>BE>BD. In contrast, N_mass_ showed the opposite trend, with NE<BE<BD. Principal components analysis (PCA) of the data showed that the multivariate space occupied by three PFTs was distinct (**Figure 2**). The first and second PC axes explained 74.8% and 12.8%, respectively. PC1 was strongly positively related to LT, LDMC, LMA, C/N, and N_area_, and negatively related to N_mass_ (**Figure 2**).

**Table 1:** Differences of leaf traits among different PFTs.

PFTs	LT(mm)	LDMC(mg/g)	LMA(mg/cm^2^)	C/N	N_area_(mg/cm^2^)	N_mass_(mg/g)
NE	0.52 ± 0.01a	415.73 ± 3.08a	19.40 ± 0.39a	38.76 ± 0.48a	0.26 ± 0.01a	13.55 ± 0.20c
BE	0.28 ± 0.01b	370.64 ± 5.96b	10.22 ± 0.40b	26.99 ± 0.71b	0.18 ± 0.01b	20.42 ± 0.54b
BD	0.19 ± 0.07c	314.73 ± 4.29c	5.53 ± 0.13c	21.48 ± 0.33c	0.12 ± 0.01c	23.47 ± 0.28a
Parameter	Result					
Slope-NE	0.063	10.206	2.415	2.733	0.027	-1.804
Slope-BE	0.002	1.493	0.086	0.066	0.002	-0.089
Slope-BD	-0.001	-2.745	-0.037	-0.057	-0.001	0.020
Sigma^2^	0.029	5.512	0.001	1.290	0.013	0.838

NE is needle-leaved evergreens. BE is broad-leaved evergreens. BD is broad-leaved deciduous. Different letters indicate significant differences among 3 PFTs (P<0.01). Sigma^2^ represents the standard deviation of linear mixed model.

### Correlation between leaf traits

**Figure 3:**
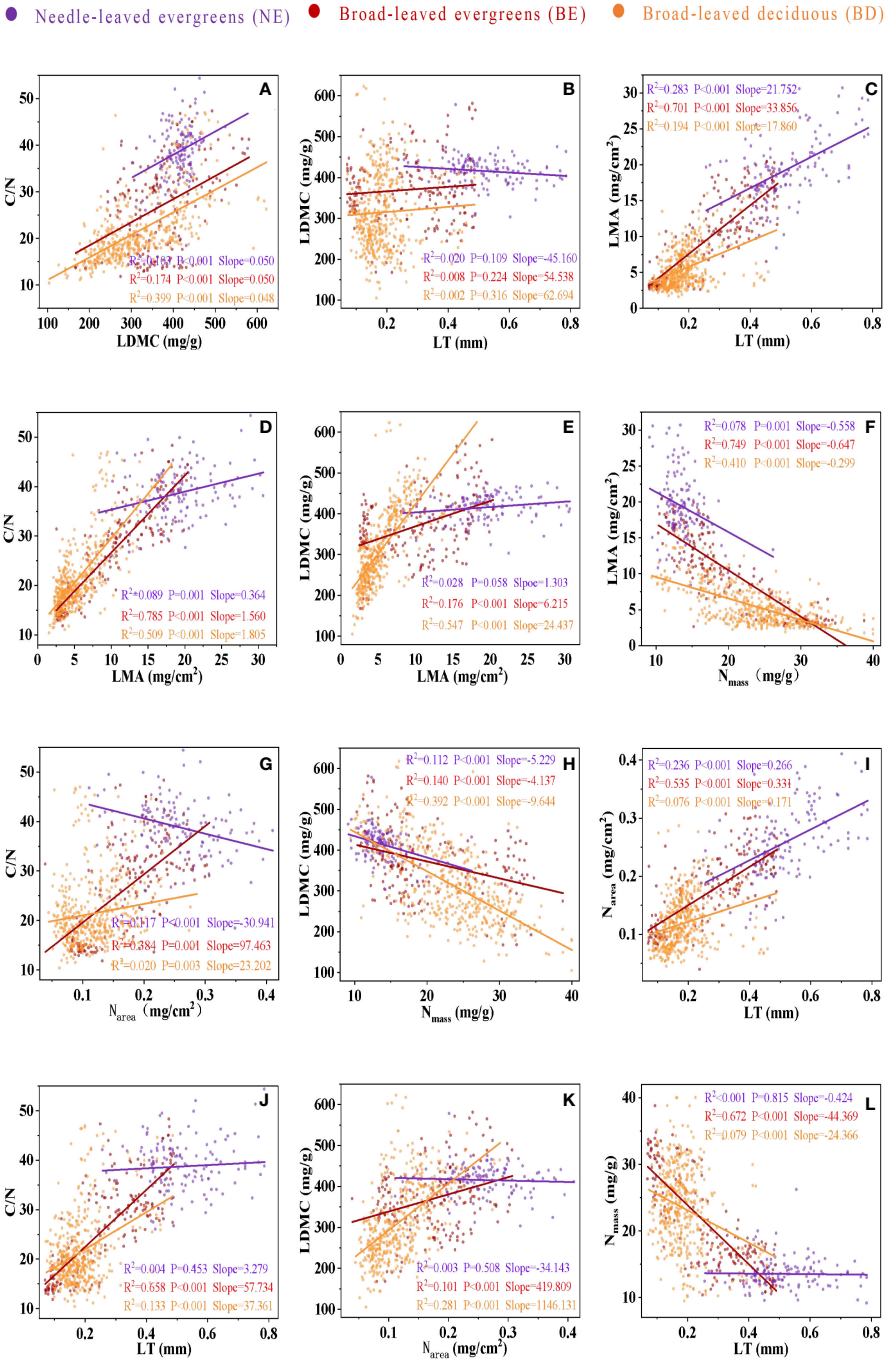
The bivariate relationship between C/N and LDMC **(A)**, between LDMC and LT **(B)**, between LMA and LT **(C)**, between C/N and LMA **(D)**, between LDMC and LMA **(E)**, between LMA and Nmass **(F)**, between C/N and Narea **(G)**, between LDMC and Nmass **(H)**, between Narea and LT **(I)**, between C/N and LT **(J)**, between LDMC and Narea **(K)**, and between Nmass and LT **(L)**. Needle-leaved evergreens (NE), broad-leaved evergreens (BE), and broad-leaved deciduous (BD) are shown in purple, red, and orange respectively. Solid lines represent the fitting curves, R2 represents the fitting degree of the solid line, P represents the significance level, and slope represents the slope of the solid line.

Significant correlations were observed between leaf traits, which varied among three PFTs (**Figure 3**; **Table 2**). In most cases, the correlations between leaf traits of three PFTs were similar. For instance, a positive correlation was observed among C/N-LDMC (**Figure 3A**), LMA-LT (**Figure 3C**), C/N-LMA (**Figure 3D**), LDMC-LMA (**Figure 3E**), N_area_-LT (**Figure 3I**), and C/N-LT (**Figure 3J**), while a negative correlation was observed between LMA-N_mass_ (**Figure 3F**), LDMC-N_mass_ (**Figure 3H**), and N_mass_-LT (**Figure 3L**). However, in only a few cases, the slope relations were significantly different among three PFTs (**Figures 3B, G, K**; **Table 2**). The common slope test showed that the scaling index varied significantly among the PFTs, indicated that the scaling relationship was dependent on the PFTs associated with most of the bivariate traits examined (**Figure 3**; **Table 2**).

**Table 2:** The result of Pearson’s correlation among different leaf traits.

PFTs	Variables	LT	LDMC	LMA	C/N	N_mass_
NE	LDMC	-0.141NS				
	LMA	0.532***	0.166NS			
	C/N	0.066NS	0.321***	0.300***		
	N_mass_	-0.021NS	-0.335***	-0.280***	-0.958***	
	N_area_	0.486***	-0.058NS	0.782***	-0.342***	-0.352***
BE	LDMC	0.091NS				
	LMA	0.837***	0.420***			
	C/N	0.811***	0.418***	0.886***		
	N_mass_	-0.820***	-0.374***	-0.865***	-0.962***	
	N_area_	0.731***	0.317***	0.886***	0.620***	-0.640***
BD	LDMC	0.047NS				
	LMA	0.441***	0.739***			
	C/N	0.364***	0.632***	0.713***		
	N_mass_	-0.280***	-0.626***	-0.641***	-0.917***	
	N_area_	0.276***	0.530***	0.757***	0.140**	-0.079NS

NE is needle-leaved evergreens. BE is broad-leaved evergreens. BD is broad-leaved deciduous. NS is not significant. ‘**’, 0.001<P ≤ 0.01. ‘***’, P ≤ 0.001.

### Relationship between leaf traits and environment

**Figure 4:**
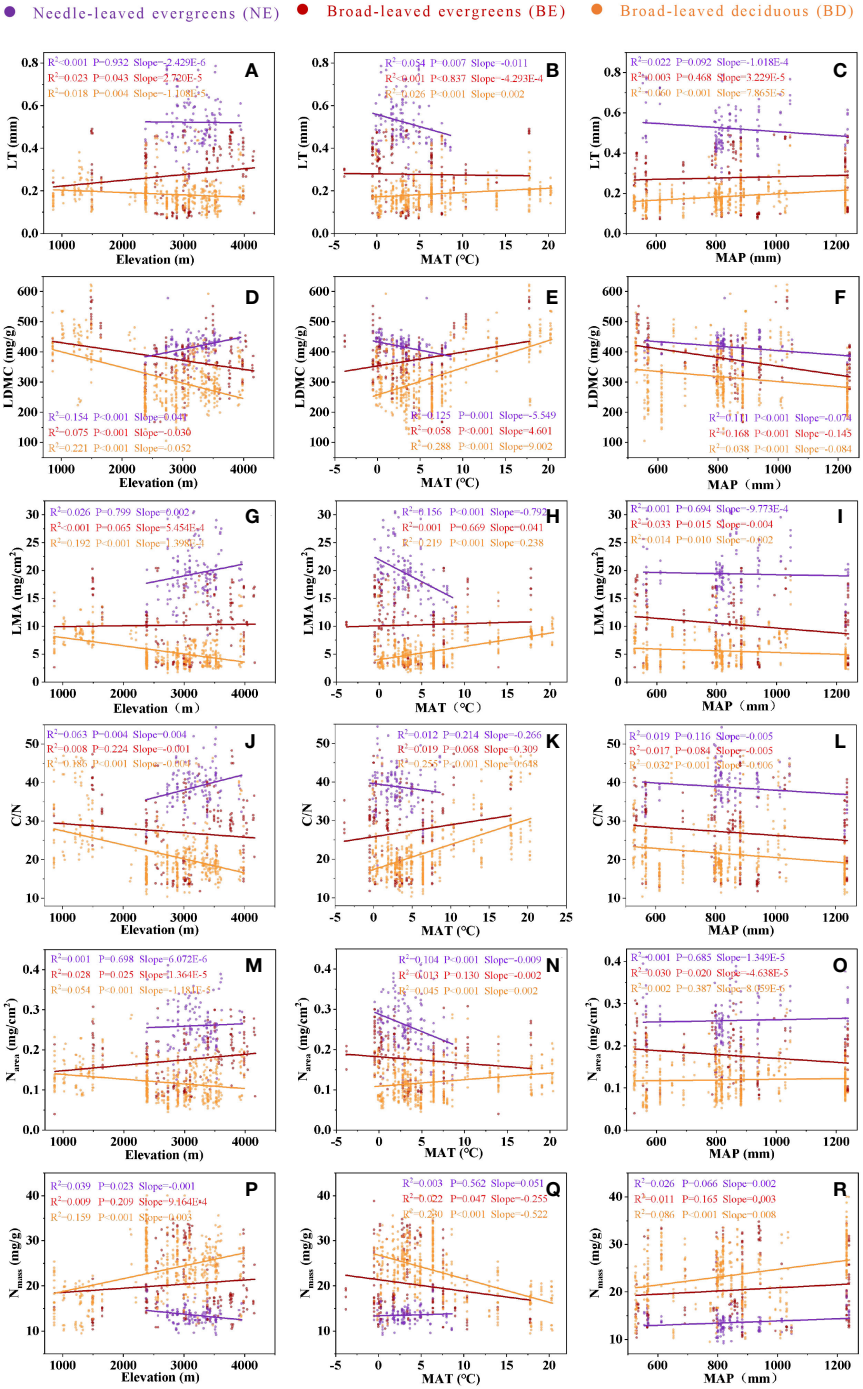
The relationship between LT and elevation **(A)**, between LT and MAT **(B)**, between LT and MAP **(C)**, between LDMC and elevation **(D)**, between LDMC and MAT **(E)**, between LDMC and MAP **(F)**, between LMA and elevation **(G)**, between LMA and MAT **(H)**, between LMA and MAP **(I)**, between C/N and elevation **(J)**, between C/N and MAT **(K)**, between C/N and MAP **(L)**, between Narea and elevation **(M)**, between Narea and MAT **(N)**, between Narea and MAP **(O)**, between Nmass and elevation **(P)**, between Nmass and MAT **(Q)**, between Nmass and MAP **(R)**. Needle-leaved evergreens (NE), broad-leaved evergreens (BE), and broad-leaved deciduous (BD) are shown in purple, red, and orange respectively. Solid lines represent the fitting curves, R2 represents the fitting degree of the solid line, P represents the significance level, and slope represents the slope of the solid line.

The analysis revealed effects of elevation, MAT, and MAP on leaf traits, and these effects varied across PFTs (**Figure 4**). While leaf traits of BE and BD exhibited similar changes with increasing elevation and MAT, NE showed a different pattern (**Figures 4A, B, D, E, G, H, J, K, M, N**). Leaf traits of three PFTs exhibited similar changes with increasing MAP, but it is not significant in most cases (**Figures 4C, F, I, L, O**).

### Comprehensive effects of PFTs, MAT, and MAP on leaf traits

**Figure 5:**
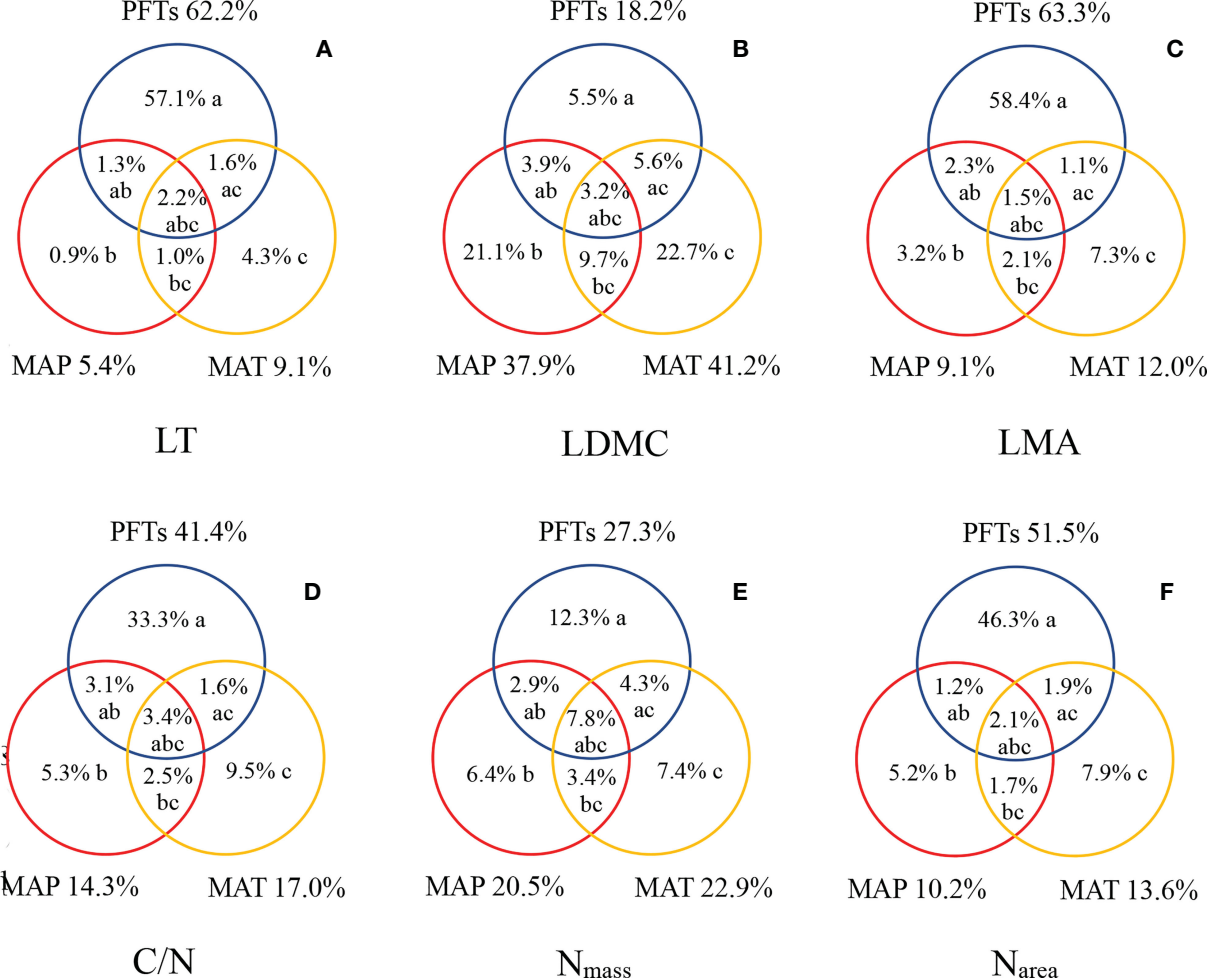
Explanation and analysis of the variations in LT **(A)**, LDMC **(B)**, LMA **(C)**, C/N **(D)**, Nmas s **(E)**, and Narea **(F)**. The symbols a, b, and c represented the independent effects of PFTs, MAP, and MAT, respectively; ab, the interactive effect of PFTs and MAP; ac, the interactive effect of PFTs and MAT; bc, the interactive effect of MAP and MAT; and abc, the interactive effect of PFTs, MAP, and MAT.

The variations in leaf traits were mainly explained by PFTs, with a higher proportion than that of MAT and MAP (**Figure 5**). The explained fractions of LT, LDMC, LMA, C/N, N_mass_, and N_area_ by PFTs were as high as 62.2%, 18.2%, 63.3%, 41.4%, 27.3%, and 51.5%. On the other hand, MAT and MAP had relatively lower explanatory power for variations of leaf traits, but had a higher proportion for LDMC and N_mass_. Compared with MAP, MAT had relatively high explanatory power (**Figures 5B, E**).

## Discussion

### Leaf traits of three PFTs

The result showed that the mean values of leaf traits differed among the three PFTs, with NE plants exhibiting greater values of LT, LDMC, LMA, C/N, and N_area_ compared to BE plants and BD plants (**Table 1**). It can be explained by NE plants’ ability to resist hostile environments, which is significantly stronger than BE plants and BD plants (Fyllas et al., 2020). The thickness of leaves can help plants avoid damage from strong light and low temperature, and it provided a protective substrate for plants to survive in hostile environment (Körner et al., 1986; Qi et al., 2014). Meanwhile, the leaf with higher LDMC had smaller intercellular space and the more gas diffusion resistance from mesophyll cells, these led to lower photosynthetic carbon assimilation capacity (Hamdani et al., 2019; Maccagni and Willi, 2022). Furthermore, according to the leaf economic theory, higher LMA indicated that NE plants adopted more conservative survival strategy, while BD plants adopted acquired survival strategy (Maracahipes et al., 2018; Fyllas et al., 2020; Rawat et al., 2021; Liu et al., 2022a). This was also confirmed in a study of woody plants from the temperate forest in the Himalayan region of India, where plants adopted conservation or acquisition strategies to adapt to environmental change (Rawat et al., 2021). The C/N and N_area_ of NE plants and BE plants were significantly higher than BD plants, while N_mass_ was lower (**Table 1**). This suggested that the nitrogen invested in the photosynthetic system of evergreen leaves (NE and BE) was relatively lower, and the remaining nitrogen was invested in non-photosynthetic systems such as cell wall proteins, lipids, and amino acids. More nitrogen was used in the construction of leaf tissue structure to make leaves tougher which enhanced the ability of evergreen plants to resist stress (Mediavilla et al., 2012; Ghimire et al., 2017; Byeon et al., 2021). On the other hand, BD plants were completely on the contrary, and they increased the nitrogen investment in the photosynthetic system so that plants with shorter leaf life could assimilate as much carbon dioxide as possible at a limited time to ensure growth and development. This indicated that there were differences in the survival strategies among three PFTs (Berveiller et al., 2007; Bai et al., 2015; Weng et al., 2017). These differences in survival strategies among the three PFTs were further confirmed by the principal component analysis (**Figure 2**), which showed differences in the multivariate space occupied by three PFTs. The smaller intersection of NE plants and BD plants indicated that they adopted different survival strategies, while BE plants were somewhere in between. The component of the first PC axis was related to the resource capture and utilization strategies of plants. It was emphasized that LMA was a better indicator of this strategy, and the dimension of resource utilization seemed to explain the more differences among PFTs (Markesteijn et al., 2011; Lasky et al., 2014). Overall, this study provided valuable insights into the survival mechanisms of plants with different functional types, which could aid in the development of effective conservation and management strategies in the future.

### Correlations among leaf traits in different PFTs

Correlations among leaf traits are essential to understand plant strategies and functional trade-offs, and these correlations are often used to infer from one trait to another in dynamic vegetation models (Lohbeck et al., 2015; Sakschewski et al., 2015). There were some similarities in the correlations among leaf traits in three PFTs (**Figure 3**; **Table 2**). LMA had significant positive correlations with LT, LDMC, C/N, and N_area_, and a negative correlation with N_mass_. However, recent studies showed that correlations among many traits might be diverse in different PFTs (Fyllas et al., 2020). For example, C/N ratio was positively correlated with N_area_ in the BE plants and BD plants, but there was a negative correlation in NE plants. The growth environment and ecological adaptation of coniferous plants and broad-leaved plants were different, which may be the reason for the different correlation between their C/N ratio and N_area_ (Ackerly and Reich, 1999; Faber et al., 2022). Coniferous plants typically thrived in nutritionally deficient soils, and they had evolved to adapt to these conditions by maintaining a high C/N ratio to more efficient use of limited nitrogen resources (Faber et al., 2022; Liu et al., 2022b). In contrast, broad-leaved plants generally grew in areas with more nutrient-rich soil, allowing them to obtain greater amounts of nitrogen during their growth and allocated it towards both growth and metabolic processes (Faber et al., 2022; Liu et al., 2022b). These differences indicated that general research phenomena might not be applicable to evaluate the correlation between leaf traits at regional or global scales in parametric dynamic vegetation models. Therefore, we suggest that PFTs-specific parameters should be developed to better represent the relationships among leaf traits that were generally embedded in such models.

### Variation in leaf traits among different PFTs along environmental gradients

The plasticity of leaf phenotype plays a crucial role in plant survival across different environments (Maire et al., 2012; Wright et al., 2017). However, it was discrepant that the sensitivity of leaves to environmental change among different PFTs (Wright et al., 2004; Kikuzawa et al., 2013). The study found that the leaf traits among three PFTs exhibited significant variation with elevation and MAT changes (**Figure 4**). As the temperature gradually decreased with increasing elevation, there was a significant collinearity between elevation and temperature (Fu and Sun, 2022). Compared with MAP, MAT was the primary environmental factor driving variation in leaf traits along elevation. With increasing elevation or decreasing temperature, the LT and N_area_ of NE plants and BE plants gradually increased, while BD plants gradually decreased. To adapt to environmental change, evergreen plants typically maintained a higher leaf thickness and internal nitrogen content at high elevations to support longer lifespans and higher photosynthetic efficiency. However, deciduous plants adopted the opposite approach to reduce nutrient and energy loss. These two PFTs took different response measures to adapt to environmental change (Niinemets, 2001; Scafaro et al., 2017; Togashi et al., 2018; Liu et al., 2022a). In addition, BD plants paid more attention to the acquisition of environmental resources in the short growing season, and weakened the investment of leaf tissue structure in environmental adaptation (Chen et al., 2013; Fyllas et al., 2020). The thinner leaves were beneficial to the gas exchange and transpiration of plants to promote the photosynthetic efficiency and enhance the carbon fixation capacity when the water was sufficient (Sun et al., 2006; Bunce, 2022). Meanwhile, with the increase of elevation, LDMC, LMA, and C/N of BE plants and BD plants showed downward trend, while N_mass_ showed upward trend. However, NE plants exhibited the opposite trend, possibly due to its tendency to adopt conservative survival strategies to adapt to environmental change, and this ensured their survival in hostile environments by enhancing leaf structure and reducing photosynthesis investment (Lasky et al., 2014). The plant with large leaf tended to prefer acquisitive survival strategies, and BD plants were the most typical representative (Fyllas et al., 2020). With the increase of MAP, the six leaf traits did not show obvious change trend, these might be because MAP was not the main environmental factor causing variation in leaf traits of woody plants in subalpine environment (Zhang et al., 2022).

PFTs, MAP, and MAT accounted for a significant portion of the biogeographic variations in leaf traits (**Figure 5**). Apart from LDMC, PFTs had a higher explanation for the variation in leaf traits. PFTs were the major factor leading to the differences in leaf traits (Akram et al., 2022). It was consistent with the results of a meta-analysis from global data conducted by Siefert et al. (2015). Compared with the MAP, MAT could explain more changes in leaf traits in subalpine environments. This finding indicated that the temperature was an important environmental factor affecting the variation in leaf traits. PFTs and climate were important drivers of variation in leaf traits, but PFTs were more critical in shaping biogeographic patterns of leaf traits, as demonstrated by previous studies from regional to global scales (Niinemets, 2001; Ordoñez et al., 2009; Zhan et al., 2018). Nevertheless, it had been proved that both soil conditions and plant phylogenetic background also affect leaf traits. Thus, further research is necessary to distinguish the relationship between the three dimensions of heredity, soil, and climate, and to explore the source of leaf traits variation caused by environmental change.

## Conclusion

There were significant differences in leaf traits among three PFTs on the eastern Qinghai-Tibetan Plateau. The result showed that NE plants, BE plants, and BD plants occupied different spatial dimensions when leaf trait was examined within all PFTs, and each PFT responded differently to environmental change (elevation and climate). Compared to mean annual precipitation (MAP), mean annual temperature (MAT) was the main environmental factor that caused the variation in leaf traits. NE plants tended to adopt a more conservative approach to survival, while BD plants were more inclined to capture and utilize current environmental resources in a large amount during a short growing season, BE plants somewhere in between. This work contributed to understanding of the regional variation in leaf traits and the relationships among leaf traits, PFT, and the environment.

## Data availability statement

The original contributions presented in the study are included in the article/**Supplementary Material**. Further inquiries can be directed to the corresponding author.

## Author contributions

HX and ZS envisioned and wrote the manuscript. SL, MC, GX, XC, MZ, JC, and FL did the experimental work, supervised by SL. All authors contributed to the article and approved the submitted version.
