# Supplementary material for: Leaf traits divergence and correlations of woody plants among the three plant functional types on the eastern Qinghai-Tibetan Plateau, China
Source: Front Plant Sci. 2023 Apr 3;14:1128227. doi: 10.3389/fpls.2023.1128227 (PMC10106608; doi:10.3389/fpls.2023.1128227)
Supplement: Supplementary file 1 [file Table_1.docx]

**Table S1** | Information about sites and collected leaf samples

| Number | Site | Vegetation types | Latitude | Longitude | Elevation | Number of species | Species name |
| --- | --- | --- | --- | --- | --- | --- | --- |
| 1 | Wenxian, Gansu Province | dry valley shrub | 32.86662222 | 104.8208139 | 861.03 | 7 | *Hibiscus syriacus*,  *Vitex trifolia*,  *Campylotropis macrocarpa*,  *Gleditsia sinensis*,  *Excoecaria acerifolia*,  *Berberis pruinosa*,  *Osteomeles schwerinae* |
| 2 | Zhouqu, Gansu Province | dry valley shrub | 33.505875 | 104.5297611 | 1496.67 | 8 | *Berberis pruinosa,*  *Ceratoideslatens*,  *Quercus spinosa*,  *Sageretia thea*,  *Zanthoxylum simulans*,  *Vitex negundo*,  *Ostryopsis davidiana*,  *Osteomeles schwerinae* |
| 3 | Zhouqu, Gansu Province | alpine shrub | 33.34744444 | 104.4966528 | 3502.79 | 1 | *Rhododendron rubiginosum* |
| 4 | Zhouqu, Gansu Province | subalpine coniferous-broadleaved mixed forest | 33.66911389 | 104.1610139 | 2730.6 | 12 | *Picea asperata*,  *Betula albosinensis*,  *Acer ukurunduense*,  *Viburnum betulifolium*,  *Fargesia nitida*,  *Abies fargesii* var. *faxoniana*,  *Lonicera japonica*,  *Philadelphus pekinensis*,  *Cornus macrophylla*,  *Mucuna sempervirens*,  *Ribes nigrum*,  *Helwingia japonica* |
| 5 | Zhouqu, Gansu Province | subalpine coniferous forest | 33.64693056 | 104.1673111 | 3336.73 | 7 | *Rhododendron rubiginosum*,  *Ribes nigrum*,  *Rosa omeiensis*,  *Rubus parvifolius*,  *Abies fargesii* var. *faxoniana*,  *Rhododendron watsonii*,  *Lonicera japonica* |
| 6 | Baishuihe, Sichuan Province | subalpine coniferous-broadleaved mixed forest | 31.33636111 | 103.8425556 | 2909.13 | 5 | *Tsuga chinensis*,  *Cerasus clarofolia*,  *Betula utilis*,  *Acer erianthum*,  *Fargesia denudata* |
| 7 | Baishuihe, Sichuan Province | subalpine coniferous forest | 31.342325 | 103.8428889 | 3207.50 | 2 | *Rhododendron rubiginosum*,  *Abies fargesii var. faxoniana* |
| 8 | Emei Mountain, Sichuan Province | alpine shrub | 29.516325 | 103.3306028 | 2998.43 | 2 | *Rhododendron rubiginosum*,  *Fargesia spathacea* |
| 9 | Emei Mountain, Sichuan Province | subalpine coniferous-broadleaved mixed forest | 29.53952778 | 103.328525 | 2378.95 | 27 | *Betula utilis*,  *Corylus ferox*,  *Eucommia ulmoides*,  *Ilex cornuta*,  *Viburnum sympodiale*,  *Abies fargesii* var. *faxoniana*,  *Viburnum fordiae*,  *Sorbus koehneana*,  *Euonymus sanguineus*,  *Cerasus clarofolia*,  *Rhododendron davidii*,  *Rubus corchorifolius*,  *Acercaudatum*,  *Corylus heterophylla* var. *sutchuenensi*,  *Enkianthus chinensis*,  *Rosa omeiensis*,  *Lonicera webbiana*,  *Rubus parvifolius*,  *Lindera glauca*,  *Padus obtusata*,  *Sorbus folgneri*,  *Smilax aberrans*,  *Meliosma cuneifolia*,  *Philadelphus pekinensis*,  *Euonymus alatus*,  *Celtis biondii*,  *Viburnum dilatatum* |
| 10 | Emei Mountain, Sichuan Province | subalpine coniferous forest | 29.51353611 | 103.3306722 | 3003.51 | 4 | *Rhododendron rubiginosum*,  *Abies fabri*,  *Fargesia spathacea*,  *Cerasus clarofolia* |
| 11 | Fengtongzhai, Sichuan Province | alpine shrub | 30.776125 | 102.67848 | 3783.30 | 2 | *Rhododendron rubiginosum*,  *Rhododendron phaeochrysum* |
| 12 | Fengtongzhai, Sichuan Province | subalpine coniferous-broadleaved mixed forest | 30.67196111 | 102.6706278 | 3122.14 | 5 | *Betula utilis*,  *Fargesia spathacea*,  *Abies fargesii* var. *faxoniana*,  *Ribes orientale*,  *Rhododendron adenogynum* |
| 13 | Fengtongzhai, Sichuan Province | subalpine coniferous forest | 30.75465278 | 102.6757861 | 3614.46 | 5 | *Rhododendron rubiginosum*,  *Rosa omeiensis*,  *Abies fargesii* var. *faxoniana*,  *Ribes orientale*,  *Lonicera tangutica* |
| 14 | Gonggangling, Sichuan Province | dry valley shrub | 33.34055833 | 104.0894694 | 1652.99 | 2 | *Berberis pruinosa*,  *Salix matsudana* |
| 15 | Gonggangling, Sichuan Province | alpine shrub | 33.039675 | 103.7180028 | 3544.29 | 2 | *Rhododendron nivale*,  *Sibiraea angustata* |
| 16 | Gonggangling, Sichuan Province | subalpine coniferous-broadleaved mixed forest | 33.33457778 | 103.7507611 | 2895.28 | 10 | *Ribes nigrum*,  *Hydrangea bretschneideri*,  *Betula albosinensis*,  *Fargesia nitida*,  *Acer davidii*,  *Tsuga chinensis*,  *Cerasus clarofolia*,  *Rubus parvifolius*,  *Acer oliverianum*,  *Sorbus koehneana* |
| 17 | Gonggangling, Sichuan Province | subalpine coniferous forest | 33.04635833 | 103.7290333 | 3567.50 | 4 | *Rhododendron williamsianum*,  *Rosa omeiensis*,  *Ribes glaciale*,  *Sorbus koehneana* |
| 18 | Hailuogou, Sichuan Province | alpine shrub | 29.5447 | 101.966475 | 3790.00 | 2 | *Rhododendron nivale*,  *Rhododendron radendum* |
| 19 | Hailuogou, Sichuan Province | subalpine coniferous-broadleaved mixed forest | 29.59041 | 102.037142 | 2537.99 | 16 | *Fargesia spathacea*,  *Acer ukurunduense*,  *Lindera rubronervia*,  *Tetracentron sinense*,  *Rhododendron delavayi*,  *Ligustrum lucidum*,  *Euonymus alatus*,  *Ilex cornuta*,  *Picea brachytyla*,  *Dlender Acanthopanax*,  *Acer*,  *Sorbus pohuashanensis*,  *Eurya alata*,  *Viburnum nervosum*,  *Maddenia wilsonii Koehne*,  *Cerasus clarofolia* |
| 20 | Hailuogou, Sichuan Province | subalpine coniferous forest | 29.574563 | 101.999707 | 3001.62 | 12 | *Abies fabri*,  *Clematoclethra lasioclada*,  *Catalpa ovata*,  *Fargesia spathacea*,  *Lonicera japonica*,  *Rhododendron rubiginosum*,  *Viburnum nervosum*,  *Euonymus alatus*,  *Viburnum dilatatum*,  *Rosa rugosa*,  *Ribes tenue*,  *Rhododendron trichanthum* |
| 21 | Maerkang, Sichuan Province | dry valley shrub | 31.64168 | 102.056095 | 2225.10 | 2 | *Rosa soulieana*,  *Sophora davidii* |
| 22 | Maerkang, Sichuan Province | subalpine coniferous forest | 31.904798 | 102.036342 | 2530.05 | 1 | *Abies fargesii* var*. faxoniana* |
| 23 | Maoxian, Sichuan Province | dry valley shrub | 31.825108 | 103.711545 | 2116.48 | 1 | *Berberis pliretii* |
| 24 | Miyaluo, Sichuan Province | alpine shrub | 31.871142 | 102.74144 | 3928.93 | 1 | *Sibiraea angustata* |
| 25 | Miyaluo, Sichuan Province | alpine shrub | 31.852051 | 102.672217 | 4148.40 | 1 | *Rhododendron aganniphum* |
| 26 | Miyaluo, Sichuan Province | subalpine coniferous-broadleaved mixed forest | 31.770777 | 102.846599 | 3369.86 | 6 | *Betula albosinensis*,  *Lonicera lanceolata*,  *Ribes nigrum*,  *Abies fargesii* var*. faxoniana*,  *Rosa omeiensis*,  *Sorbus koehneana* |
| 27 | Miyaluo, Sichuan Province | subalpine coniferous-broadleaved mixed forest | 31.78361 | 102.860768 | 3481.23 | 5 | *Betula albosinensis*,  *Rosa omeiensis*,  *Abies fargesii* var. *faxoniana*,  *Taxus cuspidata*,  *Lonicera lanceolata* |
| 28 | Miyaluo, Sichuan Province | subalpine coniferous-broadleaved mixed forest | 31.611482 | 102.860645 | 3443.30 | 5 | *Betula utilis*,  *Sorbus koehneana*,  *Abies fargesii* var. *faxoniana*,  *Ribes nigrum*,  *Rosa omeiensis* |
| 29 | Miyaluo, Sichuan Province | subalpine coniferous-broadleaved mixed forest | 31.618065 | 102.851468 | 3121.63 | 4 | *Betula albosinensis*,  *Sorbus koehneana*,  *Abies fargesii* var. *faxoniana*,  *Betula utilis* |
| 30 | Miyaluo, Sichuan Province | subalpine coniferous-broadleaved mixed forest | 31.65021 | 102.776075 | 3302.66 | 7 | *Betula albosinensis*,  *Sorbus koehneana*,  *Lonicera japonica*,  *Abies fargesii* var. *faxoniana*,  *Betula utilis*,  *Rosa omeiensis*,  *Ribes nigrum* |
| 31 | Miyaluo, Sichuan Province | subalpine coniferous-broadleaved mixed forest | 31.584457 | 102.797686 | 2915.21 | 6 | *Abies fargesii* var. *faxoniana*,  *Tsuga chinensis*,  *Betula albosinensis*,  *Sorbus koehneana*,  *Smilax chinav*,  *Acer davidii* |
| 32 | Miyaluo, Sichuan Province | subalpine coniferous forest | 31.598403 | 102.802097 | 3346.61 | 5 | *Ribes nigrum*,  *Abies fargesii* var. *faxoniana*,  *Rhododendron simsii*,  *Rosa omeiensis*,  *Lonicera japonica* |
| 33 | Miyaluo, Sichuan Province | subalpine coniferous forest | 31.871283 | 102.739489 | 3944.89 | 4 | *Rhododendron simsii*,  *Ribes nigrum*,  *Abies fargesii* var. *faxoniana*,  *Sorbus koehneana* |
| 34 | Miyaluo, Sichuan Province | subalpine coniferous forest | 31.594859 | 102.930976 | 2774.79 | 1 | *Picea asperata* |
| 35 | Mugecuo, Sichuan Province | dry valley shrub | 30.087341 | 102.176246 | 1432.09 | 2 | *Broussonetia papyrifera*,  *Vitex negundo* |
| 36 | Mugecuo, Sichuan Province | alpine shrub | 30.223191 | 101.761137 | 4036.84 | 1 | *Rhododendron lapponicum* |
| 37 | Mugecuo, Sichuan Province | subalpine coniferous-broadleaved mixed forest | 30.270447 | 101.863103 | 3166.37 | 7 | *Picea asperata*,  *Betula platyphylla*,  *Salix oritrepha*,  *Lonicera japonica*,  *Abies fabri*,  *Cerasus clarofolia*,  *Catalpa ovata* |
| 38 | Mugecuo, Sichuan Province | subalpine coniferous forest | 30.186565 | 101.875957 | 3406.47 | 7 | *Ribes tenue*,  *Abies fabri*,  *Rhododendron watsonii*,  *Lonicera japonica*,  *Rhododendron amesiae*,  *Catalpa ovata*,  *Fargesia spathacea* |
| 39 | Sandagu, Sichuan Province | subalpine coniferous-broadleaved mixed forest | 32.27656944 | 102.8639389 | 3094.43 | 6 | *Betula albosinensis*,  *Fargesia nitida*,  *Abies fargesii* var. *faxoniana*,  *Acer tetramerum*,  *Hydrangea bretschneideri*,  *Philadelphus pekinensis* |
| 40 | Sandagu, Sichuan Province | subalpine coniferous forest | 32.259558 | 102.808792 | 3527.71 | 4 | *Ribes orientale,*  *Abies fargesii* var. *faxoniana*,  *Betula utilis*,  *Salix hypoleuca* |
| 41 | Wolong, Sichuan Province | alpine shrub | 30.926478 | 102.886085 | 4219.61 | 1 | *Rhododendron aganniphum* |
| 42 | Wolong, Sichuan Province | alpine shrub | 30.898258 | 102.981832 | 3415.00 | 1 | *Sibiraea angustata* |
| 43 | Wolong, Sichuan Province | subalpine coniferous-broadleaved mixed forest | 30.854574 | 102.970794 | 2819.82 | 3 | *Betula utilis*,  *Abies fargesii* var. *faxoniana*,  *Arundinaria faberi* |
| 44 | Wolong, Sichuan Province | subalpine coniferous forest | 30.850039 | 102.973227 | 3097.55 | 2 | *Abies fargesii* var. *faxoniana*,  *Arundinaria faberi* |
| 45 | Yakexia, Sichuan Province | alpine shrub | 32.22121111 | 102.5869528 | 3987.16 | 5 | *Rhododendron nivale*,  *Sibiraea angustata*,  *Salix cupularis*,  *Rhododendron phaeochrysum*,  *Sorbus koehneana* |
| 46 | Huaping, Yunnan | dry-hot valley shrub | 26.36639 | 101.234595 | 1276.47 | 3 | *Phyllanthus emblica*,  *Jatropha curcas*,  *Dodonaea viscosa* |
| 47 | Luquan, Yunnan Province | dry-hot valley shrub | 26.305134 | 102.636237 | 1007.90 | 3 | *Dodonaea viscosa*,  *Calamus tetradactylus*,  *Vitex negundof. laxipaniculata* |
| 48 | Yongren, Yunnan Province | dry-hot valley shrub | 26.395279 | 101.460186 | 1486.32 | 3 | *Lespedezae cuneatae*,  *Quercus franchetii*,  *Dodonaea viscosa* |
| 49 | Yongsheng, Yunnan Province | dry-hot valley shrub | 26.200673 | 100.61551 | 1284.16 | 2 | *Dodonaea viscosa*,  *Vitex negundof. laxipaniculata* |
| 50 | Yuanmou, Yunnan Province | dry-hot valley shrub | 25.723384 | 101.77597 | 1211.67 | 2 | *Dodonaea viscosa*,  *Phyllanthus emblica* |
